# Melan-A/MART-1 immunity in a EWS-ATF1 translocated clear cell sarcoma patient treated with sunitinib: a case report

**DOI:** 10.1186/s12885-015-1044-0

**Published:** 2015-02-14

**Authors:** Marcella Tazzari, Elena Palassini, Barbara Vergani, Antonello Villa, Francesca Rini, Tiziana Negri, Chiara Colombo, Flavio Crippa, Carlo Morosi, Paolo G Casali, Silvana Pilotti, Silvia Stacchiotti, Licia Rivoltini, Chiara Castelli

**Affiliations:** 1 Department of Experimental Oncology and Molecular Medicine, Unit of Immunotherapy of Human Tumours, Milan, Italy; 2 Department of Cancer Medicine, Adult Sarcoma Medical Oncology Unit, Milan, Italy; 3 Consorzio MIA (Microscopy and Image Analysis), University of Milano-Bicocca, Milan, Italy; 4 Department of Diagnostic Pathology and Laboratory, Laboratory of Experimental Molecular Pathology, Milan, Italy; 5 Department of Surgery, Milan, Italy; 6 Radiology, Milan, Italy; 7 Fondazione IRCCS Istituto Nazionale dei Tumori, Via G. Venezian 1, Milan, 20133 Italy

**Keywords:** Sarcoma, Sunitinib, Clear cell sarcoma, Tumor-specific T cell, Immunotherapy, Chemotherapy

## Abstract

**Background:**

Clear cell sarcoma (CCS), initially named malignant melanoma of soft parts, is an aggressive soft tissue sarcoma (STS) that, due to MITF activation, shares with melanoma the expression of melanocyte differentiation antigens. CCS is poorly sensitive to chemotherapy. Multi-kinase inhibitors have been used as therapeutic agents. In the case we report here, treatment with sunitinib induced a long-lasting clinical response that was associated with an immune activation directed against Melan-A/MART-1 antigen.

**Case presentation:**

A 28 years old female patient with an advanced molecularly confirmed CCS resistant to conventional chemotherapy was started in January 2012 on sunitinib, 37.5 mg/day, with evidence of radiologic and metabolic response at the primary and metastatic sites of disease. Pathologic response and loss of the Melan-A/MART-1 antigen were evidenced on residual tumor removed in April 2012. Immunological monitoring performed on patient’s blood during pharmacological treatment revealed a systemic, Melan-A/MART-1 specific immunity and a low frequency of immunosuppressive cells. Sunitinib was restarted in May 2012, with a new response, and continued for 11 months although with repeatedly interruptions due to toxicity. Disease progression and new responses were documented at each treatment interruption and restart. Sunitinib was definitively interrupted in April 2013 for disease progression.

**Conclusion:**

The analysis of this case proves that antigens expressed by CCS, as for melanoma, can be immunogenic *in vivo* and that tumor-antigen specific T cells may exert anti-tumor activity in CCS patient. Thus, manipulation of the immune response may have therapeutic potential for this STS subtype and immunotherapy approaches, can be promising therapeutic options for these patients.

## Background

Clear cell sarcoma (CCS) is a very rare and aggressive soft tissue sarcoma (STS), usually arising from deep soft tissue or viscera [1], and marked by a very high metastatic risk resulting in a 5-year overall survival of about 50% [2-4]. In contrast with other STS, and similarly to melanoma, its metastatic sites include lymph nodes (LNs). CCS, initially named malignant melanoma of soft parts [5], are molecularly characterized in most cases by a specific translocation, t(12;22)(q13;q12), which results in fusion of the Ewing’s sarcoma gene, EWS, with the cyclic AMP (cAMP) regulated transcription factor, ATF1, a member of the cAMP-responsive element binding protein (CREB) family [6]. The EWS-ATF1 chimeric fusion protein interacts with the MITF (melanocyte master transcription factor) promoter, thus it directly and aberrantly activates MITF expression. Consequently, CCS is characterized by the expression of the melanocytic differentiation markers HMB-45/gp100 and Melan-A/MART-1 [7]. Overall, several immunophenotypic and molecular features are shared between CCS and malignant melanoma. Importantly, a proportion of CCS cases lack specific translocation and thus, clinical presentation as well as fluorescence in situ hybridization (FISH) analysis and reverse transcription polymerase chain reaction (RT-PCR) for the specific translocation are crucial to distinguish the two entities. Receptor tyrosine kinase expression/activation [8] and gene expression analysis [9] indicate that MITF drives the same down-stream pathways in CCS and in melanoma, and that PDGFRβ and c-Met are expressed by CCS [10,11]. Moreover, BRAF activating mutations have been occasionally detected in both EWS-ATF1 positive and negative CCS [8,12,13]. CCS is poorly sensitive to chemotherapy and anecdotal responses to regimens containing dacarbazine, vincristine, anthracycline, cyclophosphamide and to interferon-alpha-2b [14] have been reported. Based on the molecular features described above, multi-kinase inhibitors have been used as therapeutic agents in this STS and objective responses to sunitinib, and sorafenib treatments have been recently reported [15,16]. Here we describe a case of a 28 years old female patient with a metastatic, translocated CCS who experienced a prolonged, objective response to sunitinib malate (hereafter reported as sunitinib). We consider this case of interest as objective response to sunitinib correlated with a low frequency of immunosuppressive cells in the periphery, the presence of a systemic immunity directed against the CCS associated antigen Melan-A/MART-1 and the *in vivo* immune selection of post-sunitinib, MART-1 negative tumor. The analysis of this case proves that antigens expressed by CCS, as for the melanoma, can be immunogenic *in vivo* and that tumor-antigen specific T cells may exert anti-tumor activity *in vivo*.

## Case presentation

**Figure 1:**
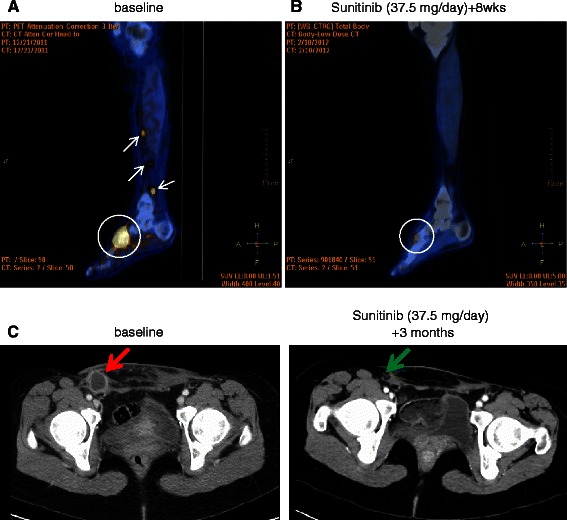
**Response to sunitinib: FDG-PET and CT scan evaluation. (A)** Baseline FDG PET/CT study: sagittal fused PET/CT image showing abnormal FDG uptakes in the left foot tumor (circle; SUVmax 12.0) and in soft tissue metastatic nodules in the ankle and leg (arrows); **(B)** After 8 weeks of treatment with SM 37.5 mg/day, PET/CT re-evaluation shows a complete metabolic response of the foot lesion (circle; SUVmax 2.5; ΔSUV −79%) and the disappearance of the soft tissue nodules. **(C)** CT scan (venous phase after contrast medium) shows a complete response to a right inguinal lymph node after 3 months of treatment with sunitinib (green arrow), compared to baseline (red arrow).

A female patient aged 28 years presented in 2007 with a lesion arising from the deep soft tissue of the left foot, covered by a healthy skin. Prior clinical history was negative for melanoma. TNM classification at presentation was: stage 3, e.g. primary lesion arising from soft tissue of the left foot with positivity of the ipsilateral inguinal sentinel LNs. On whole body CT scan there was no evidence of secondary lesions. Initial treatment consisted of wide excision of the primary tumor (surgery 2007) with diagnosis of clear cell sarcoma (CCS), and confirmed by the positivity of the FISH analysis for EWS-ATF1. The patient received sequential hyperthermic limb perfusion with melphalan and TNF and the surgical dissection of the left inguinal-iliac-obturator LNs. Three of the LNs removed were positive for disease. Local recurrence and inguinal LN relapse was detected in July 2011 and treated with doxorubicin plus dacarbazine for 5 cycles with response. Given the evidence of a new disease progression (a loco-regional and inguinal LN relapse, as confirmed by CT scan and PET) and based on preliminary evidence of sunitinib possible activity in CCS [15], in January 2012 sunitinib was started at the dose of 37.5 mg/day, with a tumor partial response (classified according to RECIST, version 1.1 [17]) to the lesion located on left foot and a complete response to metastasis on upper left leg. The response was confirmed by PET and CT scan (Figure 1). In April 2012, patient underwent left leg amputation, with evidence of pathologic response to sunitinib in the surgical specimen. In May 2012, sunitinib was restarted and maintained at the same dosage till January 2013. During these months of treatment, sunitinib was repeatedly stopped due to toxicity, with evidence of rapid disease progression following treatment interruption and of a new response after restoring treatment. From January 2013, sunitinib was reduced to 12.5 mg/day due to grade 3 cardiac toxicity. After initial disease stabilization, disease progression occurred marked by a re-growth of previously responsive tumor lesions and by the evidence of new lesions to the soft tissues of the left leg and pelvic LNs. Sunitinib was definitively interrupted in April 2013. Patient died of disease in February 2014.

**Figure 2:**
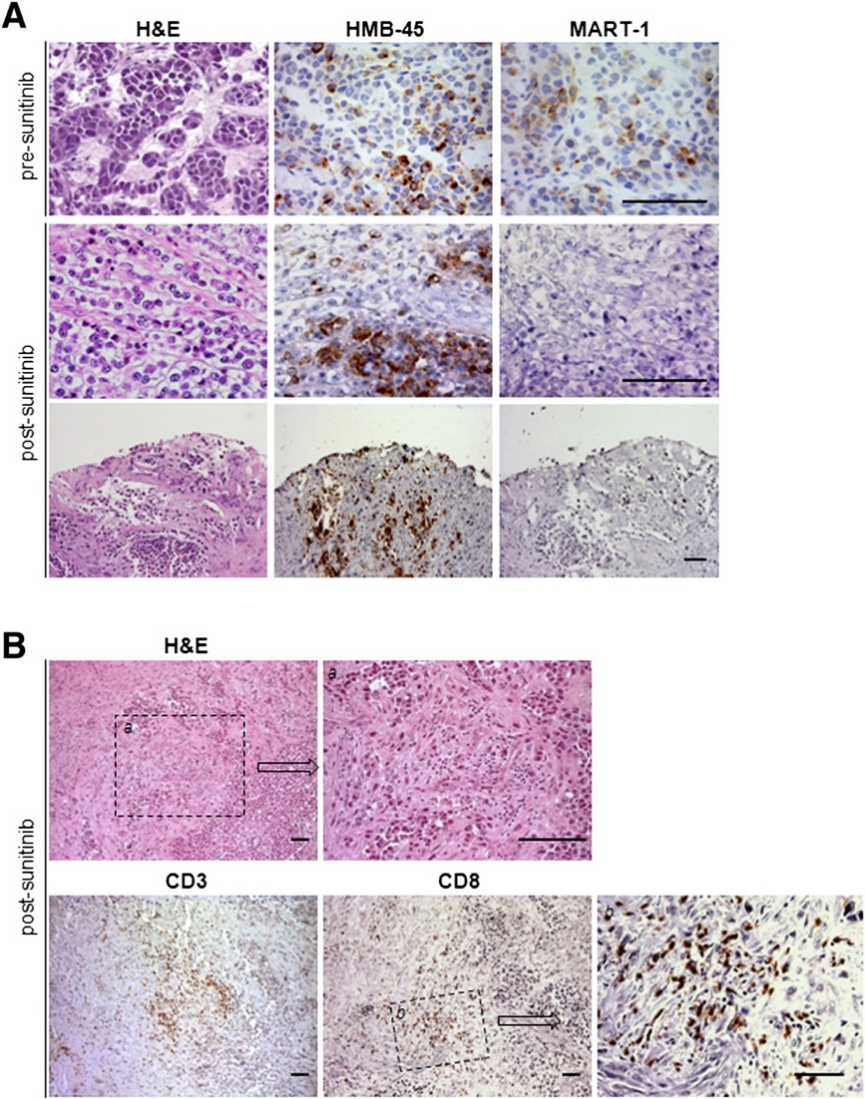
**Immunohistochemical analysis of tumor antigens expression and T cell infiltration. (A)** Hematoxylin and eosin (H&E), Melan-A/MART-1 and HMB-45/gp100 stainings in pre- (November 2011) and post- (April 2012) sunitinib tumor lesions. **(B)** Analysis of infiltrating immune T cells (CD3 and CD8) in sunitinib-treated tumor. Higher magnification in *a* shows area of pathologic tumor regression associated with lymphocyte infiltration; Bottom panels show CD3 and CD8 stainings; square *b* reports the high magnification of area infiltrated by CD8^+^ T cells. All scale bars indicate 50 μm.

**Figure 3:**
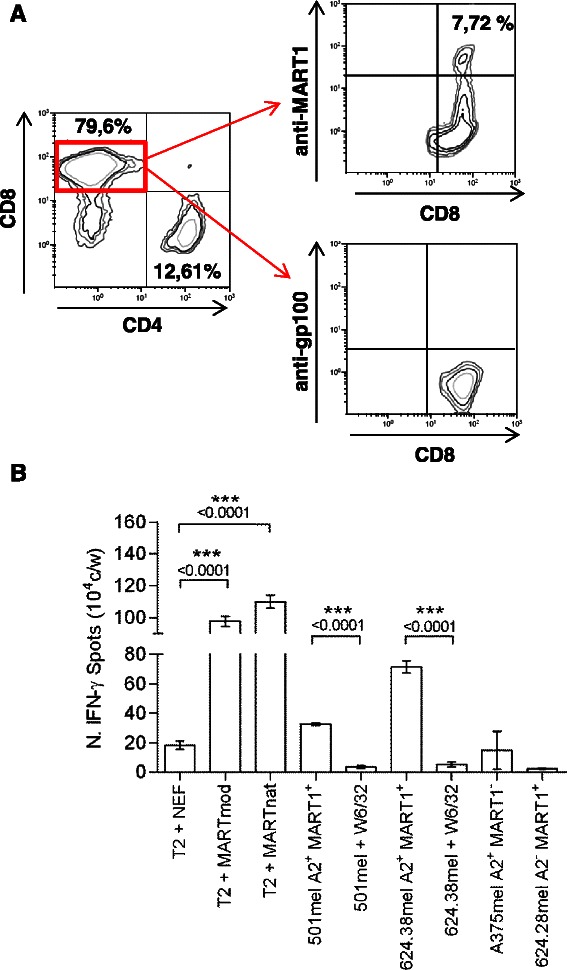
**Phenotypic and functional analysis of tumor antigen-specific CD8 T cells. (A)** Phenotypic analysis of pentamer^+^ CD8^+^ T cells after sensitization with the HLA2-A*0201 restricted-modified peptides (Melan-A/MART-1_[27L]_ or gp100_[210M]_). **(B)** The tumor specificity of peptide sensitized T cells was assessed by measuring IFN-γ secretion (Enzyme-Linked ImmunoSpot (ELISpot) assay) upon stimulation with HLA-A*0201-restricted Melan A/MART-1 (modified or native)-pulsed (2 μmol/L) lymphoblastoid T2 cell line or HLA-matched HLA-A*0201^+^MART1^+^ tumor cells (#501mel and #624.38mel) pretreated or not with the anti-HLA class I (W6/32) mAb. Moreover, T cells were also incubated with HLA-mismatched allogeneic HLA-A*0201^−^MART1^+^ (#624.28mel) or HLA-A*0201^+^MART1^−^ melanoma cells (#A375mel). The irrelevant peptide NEF_[180–189]_ was used as negative control. P values were calculated by two-tailed *t* test.

**Figure 4:**
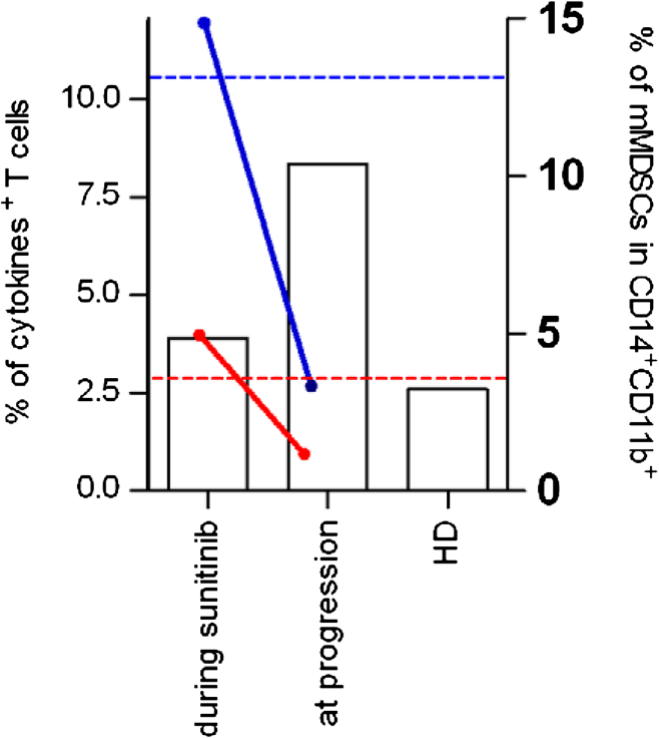
**Frequency of circulating mMDSCs and T cell function during sunitinib treatment.** Histograms show the frequencies of CD14^+^HLADR^neg/low^ (mMDSCs) in live gated CD14^+^CD11b^+^ cells of patient’s PBMCs. Average level of mMDSC frequency of healthy donors (HD) is reported. Patient’s PBMCs were assayed for IFN-γ (red line) and IL-2 (blue line) production in response to overnight activation with anti-CD3/CD28-coated beads. Dotted horizontal lines (IFN-γ (red) and IL-2 (blue)) indicate the average level of cytokine-producing T cells in HD.

Immune-related analysis were performed at the tumor site and in the peripheral blood of this patient (Table 1). The expression of the MITF regulated melanocytic antigens (HMB-45/gp100 and Melan-A/MART-1, Figure 2A) and S-100 (data not shown) was assessed by immunohistochemistry on pre- (surgery December-2010 and November-2011) and post-sunitinib tumor specimens (surgery April-2012). Pre-treatment tumor lesions displayed a clear positivity for all of the analyzed antigens. Conversely, tumor specimen removed after treatment with sunitinib displayed a selective loss of MART-1 expression, while it retained the positivity for HMB-45 and S-100 (Figure 2A). Post-sunitinib tumor was heavily infiltrated by CD3^+^ T cells that contained a significant proportion of CD8^+^ T cells. Areas of pathological regression were clearly evident in association with lymphocyte infiltration (Figure 2B). No T cells infiltration were detected in the pre-treated lesion (data not shown). The *in vivo* generation of the MART-1 loss antigen variant paralleled the presence of anti-MART-1 systemic immunity in the blood of this CCS patient. Patient’s peripheral blood mononuclear cells (PBMCs) isolated in the course of sunitinib treatment and before surgery (surgery April-2012), sensitized *in vitro* with the immunogenic HLA-A*0201 restricted peptide Melan-A/MART-1_[27L]_ displayed the presence of a remarkable frequency of MART-1 specific CD8^+^T cells (7,72%), as monitored by pentamer staining (Figure 3). These anti-MART-1 specific T cells were functionally active. MART-1 sensitized PBMC released IFNγ when stimulated with the target cells loaded with Melan-A/MART-1-epitope (modified and native) and, importantly, they recognized in a MHC restricted fashion HLA-A*0201^+^MART1^+^, but not HLA-A*0201^+^MART1^−^ and HLA-A*0201^−^MART1^+^ tumor cells as evaluated by ELIspot assay (Figure 3). Conversely, no T cells specific for the HLA-A*0201- gp100_[210M]_ peptide were detected in post-sunitinib PBMCs of the patient applying the same procedure. All together these evidences strongly support the hypothesis that the post-sunitinib MART-1 negative tumor variant is the *in vivo* outcome of a T cell-mediated immune selection occurring in CCS patient during sunitinib treatment. The anti-MART-1 systemic immunity in post-sunitinib CCS patients was associated with low frequency of circulating immunosuppressive CD14^+^CD11b^+^HLADR^neg/low^ monocytic myeloid-derived suppressor cells (mMDSCs), a population expanded in cancer patients, including melanoma [18-21]. Multi-parametric flow cytometry indicate that PBMCs collected during sunitinib treatment displayed a frequency of mMDSCs, comparable to that of healthy donors (HD) (Figure 4). Moreover, this low percentage of mMDSCs correlates with functional active convenctional T lymphocytes measured *ex vivo* as IL-2 and IFN-γ produced by CD3^+^ cells upon TCR stimulation (Figure 4). A strong increase in the number of circulating mMDSC and functionally impaired T cells was detected at the time of disease progression. Conversely, reduced frequency of CD3^+^CD4^+^CD25^hi^Foxp3^hi^ regulatory T cells (Tregs) comparable to that of HD persisted all along the drug treatment (data not shown).

**Table 1:** **Summary of the immune-related analysis**

Date		Immune-related analysis	
December 2010 (Dec-2010)	biopsy	IHC: MART-1/Melan-A +; S-100 +; gp100/HMB-45 +	
November 2011	surgery	IHC: MART-1/Melan-A +; S-100 +; gp100/HMB-45 +	
January 2012 (Jan-2012)	sunitinib		Immunological monitoring:
April 2012	surgery	IHC: MART-1/Melan-A -; S-100 +; gp100/HMB-45 +; CD3 +; CD8 +	• Frequency of immunosuppressive cells and CD3^+^ T cell function (Figure 4)
May 2012/April 2013	sunitinib*		• Presence of Melan-A/MART-1 specific CD8^+^ T cells (Figure 3)

*Abbreviations*: *IHC*, immunohistochemistry.

Note: *stopped many times due to toxicity with evidence of disease progression following treatment interruption and new response after restoring treatment.

## Conclusions

We described herein the case of a CCS (HLA-A*0201) patient with advanced disease that displayed a long-lasting response to treatment with the anti-angiogenic drug sunitinib. Based on the expression and the activation status of PDGFRβ in CSC, documented by our and other groups [8,15], sunitinib likely exerts a direct inhibition of the PDGFRβ-driven pathway in the tumor cells of the patient here studied. However, along with the effect on the tumor cells, this case report documented that in this patient, objective response during sunitinib treatment was associated to traits of tumor-specific immunity. The study of this clinical case shows that antigen expresses by CCS can be immunogenic and indicates that manipulation of the immune response may have therapeutic potential in this STS subtype. As melanoma, CCS expresses the MITF-regulated genes, including genes encoding for the melanoma differentiation antigens. Thus, we look at the presence of antigen-specific response in this CCS patient. Interestingly, we observed that tumor specimen resected after treatment with sunitinib had lost the expression of MART-1 antigen. The *in vivo* generation of MART-1 loss variant was associated to a CD3 + CD8+ T cell infiltration and to the presence of areas of pathologic regression, thus suggesting the *in vivo* occurrence of MART1-specific response. This immune contexture at the tumor site was paralleled by the finding that functionally active anti-MART-1 T cells were detectable in the blood of this patients collected during sunitinib treatment. To our knowledge this is the first report documenting the *in vivo* immunogenicity of CCS tumor. The immune response in the CSC patient studied in this report was directed toward Melan-A/MART-1. No specific immunity directed against the less immunogenic differentiation antigen gp100 was developed and, as expected, reactivity for HMB-45/gp100 was maintained in post-sunitinib surgical specimen. These findings are in line with the observation that Melan-A/MART-1/HLA-A*0201 restricted peptide behaves as immune-dominant epitope in melanoma patients and a high proportion (about 70%) of advanced stage III-IV melanoma patients display a natural anti-Melan-A/MART-1 immunity [22]. In the peripheral blood of this patient, we observed that sunitinib treatment sustained a down-modulation in the frequency of immune suppressive cells, Tregs and mMDSCs, and a parallel activation of T cell functions evaluated by the capacity of CD3^+^ T cells to release Th1 cytokines in response to a polyclonal stimulation. The immunomodulatory function of sunitinib has been clearly documented in other human tumors and we confirmed this activity in the setting of CCS [23,24]. However, our observations also suggest that the release in the immune suppression induced by sunitinib may have unleashed anti-tumor immunity in this CCS patient. Indeed, this hypothesis is in agreement with the observation that, in melanoma patients, antigen-specific responses are prevented by the presence of high frequency of circulating mMDSCs [25]. By contrast a decrease of their number favors the clinical response in patients treated with immunotherapy [26].

In conclusion, this case shed light on immune-similarities between CCS and melanoma, and indicates that manipulation of the immune response in this STS subtype likely evokes antigen-specific response. In addition to T cells specific for MITF-regulated antigens, anti-tumor immunity may potentially include also T cells recognizing unique, mutation-specific determinants. As previously shown by *in vitro* immunological assays [27], the chimeric protein encoded by the specific chromosome translocation of CCS is certainly a source for these type of antigens and it is well known that immune response directed to mutated antigens plays a crucial role in determining tumor rejection and clinical response in cancer patients under immunotherapy regimens [28,29]. Although generalized conclusion cannot be depicted from a single case, these findings suggest that immunotherapy approaches, which include tumor-specific vaccine and antibodies directed to immunological checkpoints, such as ipilimumab (anti-CTLA4) or nivolumab (anti-PD1), may offer, alone or in association with targeted-therapies, a new therapeutic option for advanced CCS patients, for which no successful therapies are currently available.

## Materials and methods

### PBMCs and cell lines

PBMCs were obtained by Ficoll density gradient centrifugation followed by cryopreservation. 501mel cell line was generated as previously described [30], 624.38mel and 624.28mel were cloned as previously described [31]. A375mel and the lymphoblastoid cell line T2 were obtained from the American Type Cell Culture (ATCC). All these cell lines were cultured in RPMI 1640 (Lonza) supplemented with 10% FCS (Lonza), Hepes and antibiotics. For tumor cell line immuno-phenotyping, the FITC–labeled BB7.2 monoclonal antibody (BD Bioscences, San Diego, CA) was used.

### Immunohistochemical analysis of antigen expression in tumor biopsies

5-μm thick formalin-fixed, paraffin-embedded tissue sections were processed for IHC staining. The monoclonal antibodies used were directed against the following antigens: anti-S100, anti-Melan-A/MART-1, anti-HMB-45/gp100, anti-CD8 (DAKO) and anti-CD3 (Novocastra).

### Lymphocyte stimulation and Enzyme-Linked ImmunoSpot (ELISpot) assay

PBMCs isolated from the patient were thawed and cultured in the presence of the HLA2-A*0201 restricted-modified peptides (Melan-A/MART-1_[27L]_ or gp100_[210M]_) (2 μmol/L) plus 60 IU/mL IL-2 (Proleukin). The cells were tested every 10 to 14 days by flow cytometry analysis for the enrichment of CD8^+^pentamer^+^ T cells. To assess their reactivity against tumor cells, IFN-γ release was determined by ELISpot assay (Mabtech) in the presence of MART1 (modified or native)-pulsed (2 μmol/L) lymphoblastoid T2 cell line or HLA-A*0201^+/−^ (MART^+/−^) melanoma cell lines. HLA class I-blocking experiments required preincubation of target cells with the W6/32 mAb.

### Flow cytometry analysis of antigen specific T cells and immunosuppressive cells

Phenotypic characterization of T cell cultures was done by the multiparametric flow cytometry analysis using the following mAbs: anti-CD8-Krome Orange (Beckman Coulter, Brea, CA), anti-CD4-APC (BD Bioscences), the HLA-A*0201 multimers were provided by Proimmune Ltd. Tregs and MDSCs frequencies were determined by multi-colour immunofluorescence staining of thawed PBMCs, excluding dead cells using the LIVE-DEAD® Fixable Violet Dead Cell Stain Kit (Life Technologies, Carlsbad, CA). For surface staining, after treatment with FcR Blocking Reagent (Miltenyi, Bergisch-Gladbach, Germany), cells were incubated with the following antibodies for 30 minutes at 4°C :APCH7-conjugated anti-CD4, PE-Cy7-conjugated anti-CD25 (for detecting Treg); APCH7 conjugated anti-CD14, PE-Cy7-conjugated anti-CD11b, PE-conjugated anti-HLADR (for detecting mMDSC). All antibodies were from BD Bioscences except PE-Cy7-conjugated anti-CD11b (from Beckman Coulter). For Treg analysis, intracellular staining with APC-conjugated anti-Foxp3 (eBioscience) or the proper isotype control (rat IgG2a) was performed. Lymphocytes activated overnight with anti-CD3/CD28 beads (DynaBeads® CD3/CD28 T cell Expander, Invitrogen Dynal AS, Oslo, Norway) in the presence of 1 μl/ml Golgi Plug (BD Biosciences) were stained for the cell surface marker CD3. The cells were then washed, fixed and permeabilized with Cytofix/Cytoperm buffer (BD Biosciences) and stained with a 488-labelled anti-IFN-γ (BioLegend), PE-labelled anti-IL-2 (BD Biosciences). Data acquisition was performed using a Gallios™ (Beckman Coulter) flow cytometer, and the Kaluza® software (Tree Star Inc, Ashland, OR) was used for data analysis.

### Consent

Written informed consent was obtained from the patient. A copy of the written consent is available for review by the Editor of this journal.
